# Prenatal detection of chromosomal abnormalities and copy number variants in fetuses with congenital gastrointestinal obstruction

**DOI:** 10.1186/s12884-022-04401-y

**Published:** 2022-01-19

**Authors:** Xinyue Meng, Lili Jiang

**Affiliations:** 1 Department of Ultrasound, Shengjing Hospital of China Medical University, Shenyang, China; 2 Department of Obstetrics and Gynecology, Shengjing Hospital of China Medical University, NO. 36, Sanhao Street, Liaoning Province 110004 Shenyang, China

**Keywords:** Congenital gastrointestinal obstruction, Copy number variation sequencing, Karyotype, Copy number variation

## Abstract

**Background:**

Congenital gastrointestinal obstruction (CGIO) mainly refers to the stenosis or atresia of any part from the esophagus to the anus and is one of the most common surgical causes in the neonatal period. The concept of genetic factors as an etiology of CGIO has been accepted, but investigations about CGIO have mainly focused on aneuploidy, and the focus has been on duodenal obstruction. The objective of this study was to evaluate the risk of chromosome aberrations (including numeric and structural aberrations) in different types of CGIO. A second objective was to assess the risk of abnormal CNVs detected by copy number variation sequencing (CNV-seq) in fetuses with different types of CGIO.

**Methods:**

Data from pregnancies referred for invasive testing and CNV-seq due to sonographic diagnosis of fetal CGIO from 2015 to 2020 were obtained retrospectively from the computerized database. The rates of chromosome aberrations and abnormal CNV-seq findings for isolated CGIOs and complicated CGIOs and different types of CGIOs were calculated.

**Results:**

Of the 240 fetuses with CGIO that underwent karyotyping, the detection rate of karyotype abnormalities in complicated CGIO was significantly higher than that of the isolated group (33.8% vs. 10.8%, *p* < 0.01). Ninety-three cases with normal karyotypes further underwent CNV-seq, and CNV-seq revealed an incremental diagnostic value of 9.7% over conventional karyotyping. In addition, the incremental diagnostic yield of CNV-seq analysis in complicated CGIOs (20%) was higher than that in isolated CGIOs (4.8%), and the highest prevalence of pathogenic CNVs/likely pathogenic CNVs was found in the duodenal stenosis/atresia group (17.5%), followed by the anorectal malformation group (15.4%). The 13q deletion, 10q26 deletion, 4q24 deletion, and 2p24 might be additional genetic etiologies of duodenal stenosis/atresia.

**Conclusions:**

The risk of pathogenic chromosomal abnormalities and CNVs increased in the complicated CGIO group compared to that in the isolated CGIO group, especially when fetuses presented duodenal obstruction (DO) and anorectal malformation. CNV-seq was recommended to detect submicroscopic chromosomal aberrations for DO and anorectal malformation when the karyotype was normal. The relationship between genotypes and phenotypes needs to be explored in the future to facilitate prenatal diagnosis of fetal CGIO and yield new clues into their etiologies.

## Background

Congenital gastrointestinal obstruction (CGIO) mainly refers to the stenosis or atresia of any part from the esophagus to the anus and is one of the most common surgical causes in the neonatal period, with an incidence of 1 in every 2000 newborns [1]. Esophageal stenosis/atresia represents a life-threatening condition in the upper gastrointestinal tract, and duodenal stenosis/atresia and jejunoileal stenosis/atresia represent the major causes of CGIO, with an incidence ranging from 1.3 to 2.8 out of 10 000 live births in the lower gastrointestinal tract [2, 3]. Colonic stenosis/atresia, the less frequent form of lower gastrointestinal tract atresia, and anorectal malformation are also included in lower gastrointestinal tract atresia. CGIO is associated with complex embryological and genetic factors. The 4–8th week of gestation in the embryonic period is known as the developmental origin of CGIO. For esophageal stenosis/atresia, a failure of invagination of the lateral trachea-esophageal grooves is proposed. Duodenal stenosis/atresia is believed to result from failure of bowel recanalization following a temporary solid stage, while the probable cause of jejunoileal stenosis/atresia and colonic stenosis/atresia may be a late mesenteric vascular accident. An impaired process in the urorectal septum might lead to anorectal malformation [4–6].

In addition to embryological factors, the concept of genetic factors as an etiology of CGIO has been generally accepted [7, 8]. The Prevention Network in the United States shows that gastrointestinal malformations are often correlated with trisomy 13, 18, 21 and Turner syndrome, in which the associations between duodenal stenosis/atresia and trisomy 21, esophageal stenosis/atresia and trisomy 18 are especially prominent [9]. With the wide application of high-resolution chromosome analysis technology in prenatal diagnosis, increasing evidence has shown that pathogenic copy number variants (pCNVs) account for a certain proportion of fetuses with ultrasound abnormalities. In recent years, chromosomal microarray analysis (CMA) has become a mature clinical high-resolution chromosome analysis technique for detecting submicroscopic chromosomal imbalances. However, the high cost and low throughput of CMA restrict its application as a routine detection method for prenatal diagnosis. With the development of next-generation sequencing (NGS) technology, NGS-based copy number variation sequencing (CNV-seq) technology has gradually developed into a high-throughput, high-resolution, short turn-around time, and low-cost detection method [10], and it has been utilized in most prenatal diagnoses as a viable alternative methodology to CMA [11, 12]. A few studies have shown that DO is also related to CNVs, such as 4q22.3 deletion [13] and 13q deletion [14, 15]. Nevertheless, genetic investigations of CGIO have mainly focused on aneuploidy, and the focus has been on DO.

Therefore, the objective of this study was to evaluate the risk of chromosome aberrations (including numeric and structural aberrations) by karyotyping in different types of CGIO. A second objective was to assess the risk of abnormal CNVs detected by CNV-seq in fetuses with different types of CGIO to provide better prenatal counseling and clinical management.

## Methods

### Subjects

We retrospectively analyzed fetuses with CGIO, alone or in combination with some soft markers and structural abnormalities, who had undergone invasive prenatal diagnosis from January 2015 to January 2020 in a tertiary care university hospital in China. Cases with failed amniocentesis or culture failure or with an abnormal family history were excluded. Our study was approved by the local Ethics Committee (approval no. 2015PS235K), and all pregnant women provided verbal consent to participate in this study via telephone.

### Ultrasonographic examination

Ultrasonographic examination was performed by two specialized sonographers using Voluson E8 or Voluson E10, Pro, Exp (GE, Milan, Italy) equipped with a 4–8 MHz transabdominal transducer. When fetal CGIO was detected, detailed anatomic scanning and fetal echocardiography were performed for each fetus. According to whether fetal CGIO was found in combination with any soft marker or other structural abnormalities, they were divided into isolated CGIO and complicated CGIO. Polyhydramnios was not included as an abnormality in this study because its development was mostly secondary to gastrointestinal obstruction.

### Cytogenetic analysis

Chromosome analysis of the fetuses was obtained using amniocentesis or cordocentesis according to the gestational weeks, which was calculated according to the last menstrual period, crown‐rump length or ultrasonographic estimation. Karyotype analysis was performed according to the G-banded karyotyping protocol on all fetal samples.

Genomic DNA (gDNA) was extracted from amniotic fluid or cord blood using the Genomic DNA Extraction Kit (Qiagen, Hilden, Germany), and then the gDNA was purified using the Purification DNA kit (Zymo Research). Invitrogen Qubit 2.0 (Thermo Fisher Scientific) was used to quantitate the concentration of gDNA. Next, the DNA library was constructed using a non‐invasive prenatal test library prep kit (Berry Genomics), in which each sample was indexed by 6 bp indexing oligos. Then, the DNA library was purified using the Purification DNA libraries for NGS kit from Berry Genomics. DNA libraries were quantitated using the Kapa SYBR fast qPCR kit (Kapa Biosystems), and the DNA standard was greater than 25 nmol/L. Then, the quantitated DNA libraries were subjected to massively parallel sequencing on the NextSeq 500 platform (Illumina), generating approximately 5 million raw sequencing reads with 36 bp genomic DNA sequences. More than 2.5 million reads were uniquely analyzed through the software provided by Berry Genomics. Several public databases, including DGV (http://projects.tcag.ca/variation), DECIPHER (http://decipher.sanger.ac.uk/), Online Mendelian Inheritance in Man (http://www.omim.org), ClinGen (https://www.clinicalgenome.org/), UCSC (http://genome.ucsc.edu/, hg19), and PubMed (http://www.ncbi.nlm.nih.gov/pubmed), were utilized to interpret the results as gains and losses of copy number. CNVs were classified as pathogenic, benign or variants of unknown significance (VOUS). According to the American College of Medical Genetics standards and guidelines for the interpretation and reporting of postnatal constitutional CNVs, the VOUS category was further subdivided into likely pathogenic, VOUS and likely benign variants [16]. In our study, only pathogenic CNVs (pCNVs), likely pCNVs and VOUS were recorded.

### Follow up

Operative reports and medical records were followed up to confirm the diagnosis of congenital gastrointestinal atresia for liveborn cases. A telephone follow-up was performed for patients who did not undergo surgery in our hospital. The development of the surviving infants was performed by trained pediatricians until 1 year old.

### Statistical analysis

Statistical analysis was performed with SPSS Statistics version 26 software (IBM, Armonk, NY, USA). Continuous variables were documented as the mean and the standard deviation (SD), and categorical variables were documented as percentages. The chi-square test or Fisher’s exact test was applied to compare the significance of differences among CGIO groups. A p value < 0.05 was considered significant.

## Results

### Subjects

A total of 240 fetuses prenatally diagnosed with CGIO were enrolled in this study from 2015 to 2020. The maternal age was 29.45 ± 4.61 years (range, 21‐43), and the gestational age at invasive testing was 27.39 ± 3.83 weeks (range, 19‐35).

### Ultrasonography findings

Of the 240 cases, there were 28 cases of esophageal stenosis/atresia, 134 cases of duodenal stenosis/atresia, 43 cases of jejunoileal stenosis/atresia, 10 cases of colonic stenosis/atresia and 25 anorectal malformations. All cases were classified into 2 groups: either isolated CGIO (69.2%, 166/240) or CGIO with other abnormalities-complicated CGIO (30.8%, 74/240). The demographic characteristics concerning isolated CGIOs and complicated CGIOs are presented in Table 1. The main accompanying abnormalities included nasal bone absence (8), single umbilical artery (12), persistent right umbilical vein (4), ventriculomegaly (5), fetal growth restriction (4), hydronephrosis (2), persistent left superior vena cava (7), tetralogy of fallot (1), pulmonic stenosis (1), ventricular septal defect (2), complete atrioventricular canal malformation (1), complete transposition of great arteries (1), renal agenesis (1), complex congenital heart disease (2), multiple echo enhancement foci in the abdominal cavity (1), gallbladder not shown (1), strephenopodia (1), shortened bones (4), echogenic bowel (1), diaphragmatocele (1), microtia (1), ventricular ependymal cysts (1), dysplasia of the septum pellucidum (2), renal cyst (1), ascites (3), situs inversus viscerum (1), cervical lymphatic hygroma (3), enlarged cisterna magna (1), vermian hypoplasia (1), situs inversus (2), polyhydramnios (92), and multiple malformations (9).

**Table 1:** Demographic characteristics of pregnancies with CGIO

Characteristic	Total (*n* = 240)	Isolated CGIO (*n* = 166)	Complicated CGIO (*n* = 74)
Maternal age (years), mean ± SD	29.45 ± 4.61	29.41 ± 4.62	29.57 ± 4.61
Gestational age at invasive testing (weeks), mean ± SD	27.39 ± 3.83	27.42 ± 3.95	27.34 ± 3.54
BMI, mean ± SD	26.3 ± 4.1	26.1 ± 3.9	26.4 ± 3.8
Parity			
Primipara, n (%)	157 (65.4)	106 (63.9)	51 (68.9)
Multipara, n (%)	83 (34.6)	60 (36.1)	23 (31.1)
Gestational count			
Singleton, n (%)	225 (93.8)	162 (97.6)	63 (85.1)
twins, n (%)	15 (6.2)	4 (2.4)	11(14.9)
Fetal gender			
Male, n (%)	122 (50.8)	96 (57.8)	26 (35.1)
Female, n (%)	118 (49.2)	70 (42.2)	48 (64.9)

*CGIO* congenital gastrointestinal obstruction, *SD* standard deviation, *BMI* body mass index

### Karyotyping

Table 2 shows the distribution and incidence rates of karyotype abnormalities in the isolated CGIO group and complicated CGIO group. The karyotype analysis of 240 fetuses with CGIO identified 43 cases with pathogenic variants, with an overall detection rate of 17.9% (43/240). Twenty-five cases of karyotype abnormalities were detected in the complicated CGIO group (74 cases), which was a significantly higher rate than that in the isolated group (25/74, 33.8% vs. 18/166, 10.8%, *p* < 0.01). Numerous chromosomal abnormalities were significantly more abundant in complicated CGIO than isolated CGIO (28.4% vs. 7.8%, *p* < 0.01); however, we observed no significant differences in structural chromosomal abnormalities or other abnormalities between complicated CGIO and isolated CGIO (both *p* > 0.05). Among these cases, numerical chromosome abnormalities were detected in 34 fetuses: trisomy 21 was found in 27 (including 2 mosaic aneuploidies); trisomy 18, trisomy13, and an aberration of the sex chromosome—47, XXY—were each detected in four, two and one cases, respectively. Structural chromosome aberrations were identified in five fetuses whose karyotypes were 46, XN, inv (9) (p11q13); 45, XN, der (13; 14) (q10; q10); 47, XN, + del (22) (q13); 46, XN, der (14), t (6;14) (q21; p11) and 46, XN, t (12;15) (q13; q21). In addition, four cases with mosaicism were also detected, including 45, XN, -13 [1] (SC)/46, XN, del (13) (q14) [1] (SC)/46, XN [42]; 46, XN, del (10) (p11-qter) [1] /46, XN [19]; 47, XN, + 2 [1] /46, XN [49] and 47, XN, + mar [33] /46, XY [9]. The rates of chromosomal abnormalities in fetuses with esophageal stenosis/atresia, duodenal stenosis/atresia, jejunoileal stenosis/atresia, colonic stenosis/atresia and anorectal malformation were 14.3% (4/28), 25.0% (33/132), 4.4% (2/45), 0 and 16.0% (4/25), respectively. The distributions and rates of chromosomal abnormalities for different types of CGIO are shown in Table 3.

**Table 2:** Rates of abnormal karyotypes of fetuses with isolated CGIO and complicated CGIO

	Total (*n* = 240)	Isolated CGIO (*n* = 166)	Complicated CGIO (*n* = 74)
Numeric			
Trisomy 21, n (%)	27 (11.3)	12 (7.2)	15 (20.3)
Trisomy 18, n (%)	4 (1.7)	0	4 (5.4)
Trisomy 13, n (%) 47, XXY Total, n (%)	2 (0.8) 1 (0.4) 34 (14.2)	0 1 (0.6) 13 (7.8)	2 (2.7) 0 21 (28.4)^a^
Structure			
Inversion, n (%)	1 (0.4)	1 (0.6)	0
Translocation, n (%)	3 (1.2)	1 (0.6)	2(2.7)
Deletion, n (%) Total, n (%)	1 (0.4) 5 (2.1)	0 2 (1.2)	1 (1.4) 3 (4.1)
Others, n (%)	4 (1.7)	3 (1.8)	1 (1.4)
Total, n (%)	43(17.9)	18 (10.8)	25 (33.8)^a^

*CGIO* Congenital gastrointestinal obstruction, ^a^Differences between isolated CGIO and complicated CGIO groups were statistically significant (*P* < 0.01)

**Table 3:** The distributions and rates of chromosomal abnormalities for different types of CGIO

	Total, n (%)	Trisomy 21	Trisomy 18	Trisomy 13	47, XXY	Inversion	Translocation	Deletion	Others
esophageal stenosis/atresia (*n* = 28)	4 (14.3)	0	3	0	0	0	Reciprocal (1)	0	0
duodenal stenosis/atresia (*n* = 132)	33 (25)	27	0	0	1	Chromosome 9 (1)	Robertsonian (1) Reciprocal (1)	0	Mosaicism (2)
jejunoileal stenosis/atresia (*n* = 45)	2 (4.4)	0	0	0	0	0	0	0	Mosaicism (2)
colonic stenosis/atresia (*n* = 10)	0 (0)	0	0	0	0	0	0	0	0
anorectal malformation (*n* = 25)	4 (16)	0	1	2	0	0	0	Chromosome 22q (1)	0
Total (*n* = 240)	43 (17.9)	27	4	2	1	1	3	1	4

### CNV-seq results

Among the 197 cases with normal karyotypes, 93 cases underwent further CNV-seq: chromosomal CNVs were found in 26 cases with a positive rate of 28.0% (26/93), of which 9 pathogenic/likely pathogenic CNVs were detected in 8 cases (9.7%, 9/93), 2 cases involved VOUS (2.2%, 2/93), and 16 cases involved benign/likely benign CNVs. The rates of pCNV, likely pCNV and VOUS in different types of CGIO are presented in Table 4, and the related Online Mendelian Inheritance in Man (OMIM) genes are shown in Table 5. CNVs of pathogenic/likely pathogenic (*n* = 9) ranged in size from 0.2 Mb to 9.76 Mb, among which were 2 cases with the deletion of 13q and 2 cases with 22q11.2 duplication. Aberrations derived from 4q comprised one case of deletion and one case of duplication. Other associated chromosome anomalies in our study consisted of 10q26, 2p24 and 20p12.2.

**Table 4:** Rates of pCNV, likely pCNV and VOUS in the different types of CGIO

	All (93)			Isolated (63)			Complicated (30)		
	N	pCNV/likely pCNV, n (%)	VOUS, n (%)	N	pCNV/likely pCNV, n (%)	VOUS, n (%)	N	pCNV/likely pCNV, n (%)	VOUS, n (%)
esophageal stenosis/atresia	12	0	0	9	0	0	3	0	0
duodenal stenosis/atresia	40	7 (17.5)	1 (2.5)	30	3 (10.0)	0	10	4 (40.0)	1 (10.0)
jejunoileal stenosis/atresia	22	0	0	17	0	0	5	0	0
colonic stenosis/atresia	6	0	0	4	0	0	2	0	0
anorectal malformation	13	2 (15.4)	1 (7.7)	3	0	0	10	2 (20.0)	1 (10.0)
Total, n (%)	93	9 (9.7)	2 (2.2)	63	3 (4.8)	0	30	6 (20.0)^a^	2 (6.7)

*pCNV* pathogenic copy number variants, *VOUS* variant of uncertain significance, *CGIO* congenital gastrointestinal atresia

^a^Differences between isolated CGIO and complicated CGIO groups were statistically significant (*P* < 0.05)

**Table 5:** Characteristics of 10 CGIOs with clinically significant CNVs or VOUS, as detected by copy number variation sequencing

Case	gestational weeks	Gastrointestinal anomaly	Associated Anomalies	CNVs	CNV type	CNV size (Mb)	OMIM genes	Associated Syndrome	Categorization	Inheritance	Outcome
1	23	duodenal stenosis/atresia	None	arr13q21.33q34(69,340,000–79,100,000) × 1	Deletion	9.76	EDNRB (131,244)	Waardenburg syndrome, type 4A (277,580)	Pathogenic	De novo	TOP
2	25	duodenal stenosis/atresia	Single umbilical artery	arr13q22.31q33.3(70,690,000–79,680,000) × 1	Deletion	8.99	EDNRB (131,244)	Waardenburg syndrome, type 4A (277,580)	Pathogenic	De novo	TOP
3	37	Anorectal malformation	No gallbladder	arr22q11.21(19,020,000–21,480,000) × 3	Gain	2.46	—	22q11duplication syndrome (608,363)	Pathogenic	De novo	TOP
4	30	duodenal stenosis/atresia	Shortened limbs	arr22q11.23(23,700,000–25,120,000) × 3	Gain	1.42	—	22q11duplication syndrome (608,363)	Likely Pathogenic	Paternal	TOP
5	32	duodenal stenosis/atresia	Persistent left superior vena cava	arr10q26.13q26. 3(125,600,000–135,440,000) × 1;	Deletion	9.84	EBF3 (607,407)	Hypotonia, ataxia, and delayed development syndrome (617,330)	Pathogenic	De novo	full-term delivery
				arr4q24(106,920,000–107,480,000) × 1	Deletion	0.56	TBCK (616,899) AIMP1 (603,605)	Hypotonia, infantile, with psychomotor retardation and characteristic facies-3 (616,900)	Pathogenic	Maternal	
								Leukodystrophy, hypomyelinating-3 (260,600)			
6	32	duodenal stenosis/atresia	None	arr4q11q13.1(52,680,000–60,420,000) × 1	Gain	7.74	—	—	Likely Pathogenic	De novo	intrauterine fetal death at 31 weeks
7	24	duodenal stenosis/atresia	None	arr2p24.3p24.2(13,900,000–18,720,000) × 1	Deletion	4.82	MYCN(164,840)	Feingold syndrome 1 (164,280)	Pathogenic	De novo	TOP
							NBAS (608,025)	Short stature, optic nerve atrophy, and Pelger-Huet anomaly (614,800) infantile liver failure syndrome-2 (616,483)			
8	23	Anorectal malformation	Renal cyst	arr20p12.2(10,360,000–11,060,000) × 1	Deletion	0.70	JAG1 (601,920)	Alagille syndrome (118,450)	Likely Pathogenic	De novo	TOP
9	26	duodenal stenosis/atresia	Absent nasal bone	arr5p15.33(820,000–1,040,000) × 3	Gain	0.22	TRIP13 (604,507)	MVA3 (617,598)	VOUS	Paternal	full-term delivery
10	23	Anorectal malformation	Lymphangioma of the neck, single umbilical artery	arr14q24.1(68,200,000–68,480,000) × 1	Deletion	0.28	ZFYVE26(612,012)	Spastic paraplegia-15 (270,700)	VOUS	Maternal	full-term delivery

*OMIM* Online Mendelian Inheritance in Man, *VOUS* variant of uncertain significance, *CNV* copy number variant, *TOP* termination of pregnancy

In the isolated CGIO group, the overall detection rate of pCNVs or likely pCNVs was 4.8% (3/63). Considering only the isolated duodenal stenosis/atresia group, pCNVs and likely pCNVs were found in 10% of cases (3/30). Meanwhile, the detection rate of pCNVs or likely pCNVs in fetuses with complicated CGIO was 20.0% (6/30), and the highest detection rate was in the duodenal stenosis/atresia group, which was 40%, followed by the anorectal malformation group (20.0%, 2/10). The overall detection rate of pCNVs and likely pCNVs in the complicated CGIO group was significantly higher than that of the isolated CGIO group (20.0% vs. 4.8%, *p* < 0.05). Concerning subtypes of CGIO, abnormal CNVs were detected in the duodenal stenosis/atresia group (17.5%, 7/40) and anorectal malformation group (15.4%, 2/13), while no abnormal CNVs were detected in the esophageal stenosis/atresia group, jejunoileum stenosis/atresia group or colonic intestine/stenosis group.

VOUS was reported in 2 cases of CGIO (2/93, 2.2%), and both were in the complicated CGIO group. One duplication of uncertain significance was identified in duodenal stenosis/atresia in the absence of a nasal bone. One deletion of uncertain significance was identified in anorectal malformation with lymphangioma of the neck and a single umbilical artery.

### Follow-up

Follow-up was obtained in 219 (91.3%, 219/240) of our patients. Forty-three fetuses either died in utero or were terminated. Among the surviving infants, 9 cases were not found to have any gastrointestinal obstruction syndrome, and the postpartum imaging was normal; 8 cases were misdiagnosed, and the postpartum results were inconsistent with the prenatal diagnosis, including 4 cases of Hirschsprung's disease, 3 cases of intestinal volvulus, and 1 case of congenital choledochal cyst. In addition, 3 infants had structural abnormalities that were not diagnosed by prenatal ultrasound, including 1 case of congenital hypoplasia of the penis, cryptorchid, and congenital cleft palate; 1 case of microtia; and 1 case of hypospadias and tethered cord syndrome. During the follow-up period, one infant with duodenal stenosis/atresia and persistent left superior vena cava with a 10q deletion (case 5) showed developmental delay, and the others showed no obvious phenotypic abnormality.

## Discussion

Congenital gastrointestinal obstruction is one of the most frequent anomalies second to central nervous system anomalies in fetuses [17]. The causes of gastrointestinal atresia are complex, and chromosomal abnormalities constitute an important factor in its pathogenesis. Studies published to date have mainly focused on the relationship between aneuploid and CGIO. Up to 44% of fetuses with duodenal stenosis/atresia are associated with trisomy 21 [18–20] and may be higher if other chromosomal abnormalities are included. In our research, even if chromosome structure abnormalities were included, the rate of abnormal karyotypes (including trisomy 21) in cases with duodenal atresia was only 25%, which was lower than that in previous studies. Becky et al. reported that only 15% (4/27) of cases with DA were diagnosed with trisomy 21, at a lower rate; the same was true in the study by Zhang et al., at a detection rate of 5.9% (3/51). Combined with our research, we believe that the association between duodenal atresia and trisomy 21 should be reconsidered because of the wide use of noninvasive prenatal testing (NIPT) and improvements in ultrasonographic techniques in the first trimester. In addition, we found that the prevalence of chromosomal anomalies in cases of isolated CGIO (10.8%) was lower than that with complicated CGIO (33.8%), consistent with that reported in previous studies [21], indicating that it is necessary to strengthen the observation of other structures or soft markers when CGIO is encountered.

Although both CMA and CNV-seq provide high-resolution analysis for accurate and reliable diagnosis of clinically significant CNVs, CNV-seq does have some potential advantages over CMA, including high throughput sample analysis, lower gDNA input detection, and shorter turnaround time. In the past few years, several large studies published the incremental yield of CMA over karyotype in fetuses with ultrasonographic structure anomalies [22–24], but few studies were conducted on the relationship between CNV-seq and karyotype. In 2018, a large prospective study that performed CNV-seq on 3429 amniotic fluid samples in fetuses with a low risk of CNV abnormalities showed that the incremental yield of CNV-seq over karyotype was approximately 1% and first proposed that CNV-seq could be considered the first-tier diagnostic technique for detecting pCNVs [11]. Moreover, it was suggested that the frequency of pCNVs in fetuses with ultrasonographic abnormalities should be further refined by the organ system involved and the number of anomalies observed [25]. Xia et al. reported that the detection rates of pCNV and VOUS were 4.55% and 9.09% in digestive disorders, respectively [26]. In a recent study performed by Zhang et al. [15], pCNVs were identified in 5 of 48 fetuses with DO at a detection rate of 10.4%. In our study, we observed different types of CGIO caused by stenosis or atresia. CNV-seq revealed an incremental diagnostic value of 9.7% over conventional karyotyping in fetuses with CGIO, and the genomic alterations differed between isolated CGIO and complicated CGIO. The incremental diagnostic yield of CNV-seq in complicated CGIOs (20%) was higher than that in isolated CGIOs (4.8%), and the highest prevalence of pCNVs/likely pCNVs was found in the duodenal stenosis/atresia group (17.5%), followed by the anorectal malformation group (15.4%). These data were inconsistent with the reports by Bishop et al. [13] in which a pathogenic microdeletion was found only in isolated duodenal atresia and by Zhang et al. [15] in which no significant difference in pCNVs was observed between the isolated group and complicated group. However, we believe that the divergence might be due to the relatively low number of fetuses included in those studies.

The 13q deletion, resulting in Waardenburg syndrome type 4A (OMIM 277580), was the most frequently identified CNV in our study (cases 1 and 2). This finding was consistent with a finding in a previous study [15]. CNV-seq revealed a 9.76-MB deletion in 13q21.33q34 and an 8.99-MB deletion in 13q22.31q33.3. According to the AMC guidelines, they were both classified as pathogenic CNVs. The ClinGen database shows that the fragment contains the EDNRB gene, which is a signaling molecule and key component of the endothelin pathway and plays an important role in the migration of enteric nervous system (ENS) precursors within the gut [27]. Several studies reported that mutations of EDNRB were identified in Hirschsprung disease (HSCR, absence of enteric neurons in distal portions of the gut) and Waardenburg syndrome type 4A (WS4A, pigmentation defects and deafness due to altered development of melanocytes) [27–30]. Notably, abnormal migration of neural crest cells in conjunction with destruction of blood vessels may be the possible pathogenesis of duodenal atresia. Meanwhile, the relationship between 13q deletion and duodenal atresia has been described in several case reports [15, 31, 32]. In summary, we consider that haploinsufficiency of EDNRB might be a candidate gene to produce the phenotype of duodenal stenosis/atresia in a fetus with 13q deletion syndrome.

Another variant that occurred with high frequency was 22q11.2 microduplication, which was present in one case of anorectal malformation (case 3) and one case of duodenal atresia (case 4). The 22q11.2 duplication is associated with mild but highly variable phenotypes, ranging from normal to developmental delay, growth restriction, hypotonia, and intellectual disability [33]. Due to incomplete penetrance and variable phenotypes, this CNV can cause parental anxiety about the future health and development of their children. Although the effects of these potentially pathogenic abnormalities do not appear after birth or for a long time after birth, the limited information on 22q11.2 duplication is not trivial. Providing parents with this information may not only help parents better understand abnormalities that may occur in their children but also help families take better care of them and seek therapeutic intervention earlier.

Case 5 was a fetus with a 9.84-Mb 10q26.13q26.3 deletion (de novo) and a 0.56-Mb 4q24 deletion (maternal) with duodenal atresia and persistent left superior vena cava on prenatal ultrasound. The deletion of 10q26 includes the EBF3 gene, of which low expression has been linked to hypotonia, ataxia, and delayed development syndrome (OMIM 617330), resulting in various congenital abnormalities, including microcephaly, growth retardation, intellectual disability, craniofacial dysmorphism, micropenis, cryptorchidism, etc. [34–37]. Maruyama et al. reported a patient with duodenal atresia combined with partial monosomy 10q with partial trisomy 11q, showing that the synergistic effects of partial monosomy 10q and partial trisomy 11q on the phenotype might be related to the development of duodenal atresia [38]. The deletion of 4q24 encompasses the TBCK and AIMP1 genes, and loss of them may be responsible for psychomotor retardation. Bishop et al. [13] reported a case of isolated duodenal atresia who had a likely pathogenic microdeletion of chromosome 4q22.3. Therefore, the literature, combined with our current results, suggests that 10q26 and 4q24 microdeletions might be additional genetic etiologies of duodenal stenosis/atresia.

Case 7, a fetus with duodenal atresia, had a 4.82-Mb deletion in chromosome 2p24.3p24.2. This segment contains the genes MYCN and NBAS. MYCN is significantly associated with Feingold syndrome-1 (FGLDS1), which is characterized by esophageal and duodenal atresia, microcephaly, limb malformation, and mental retardation. Vertebral anomalies, cardiac malformations, and deafness have also been reported in a minority of patients [39]. NBAS is associated with infantile liver failure syndrome, short stature and optic nerve atrophy [40, 41]. Although diagnosed with a normal karyotype, TOP was chosen for this patient due to pathogenic CNV. For the deletion of 20p12.2 and the duplication of 4q in our reports, there are no previous studies about their association with congenital gastrointestinal deformities and were considered to be incidental findings by us. In addition, two VOUS were detected in one case with duodenal atresia and one case with anorectal malformation accompanied by other structural abnormalities. The fetuses were both delivered at term, showing no obvious phenotypic abnormality up to the time the article was written.

The mortality rate of CGIO is usually low. Most neonates with these conditions can be relieved by surgery, and the overall outcome is good; however, the premise is that there are no genetic abnormalities in prenatal diagnosis. Submicroscopic chromosome aberrations should be considered in addition to aneuploidies. In our study, no pathogenic CNVs were found in esophageal stenosis/atresia, jejunoileum stenosis/atresia or colonic intestine/stenosis. Rare inherited and de novo CNVs were identified in fetuses with esophageal atresia. A multicenter study detected 375 patients with esophageal atresia and found that 2.7% had pathogenic CNVs, expanding the genetic scope of esophageal atresia [42]. Therefore, the correlation between esophageal atresia and CNVs still requires a large number of case studies and targeted genotype–phenotype analysis. Approximately 55% of infants born with esophageal atresia have other anomalies or birth defects, 10% of infants have a nonrandom VACTERL syndrome, and 1% of infants also have CHARGE syndrome, so fetuses with esophageal atresia require detailed imaging and genetic testing to assess the risk [43–46]. Research has suggested that the occurrence of jejunoileum stenosis/atresia is related to race and maternal age, and the correlation between them and chromosomal abnormalities is lower than that of duodenal atresia and esophageal atresia [47]. In 2015, a number of European research centers retrospectively analyzed 423 cases of jejunoileum stenosis/atresia and found that the prevalence of chromosomal abnormalities was 3.8% and that of aneuploidy was only 0.3% [21]. These findings demonstrated that the incidence of chromosomal abnormalities of jejunoileum stenosis/atresia was relatively low. The mechanism of colonic atresia is generally accepted to be vascular injury. This may be the reason for its low incidence of chromosomal abnormalities. Meanwhile, the weak connection between chromosomal abnormalities and jejunoileum stenosis/atresia and colonic intestine/stenosis observed in our study provides information for clinical prenatal counseling and decision-making.

There are several limitations in our study. First, as a retrospective study, there were limited numbers of some categories of CGIO, and less than half of the fetuses underwent CNV-seq. Therefore, the pCNV and likely pCNV rates of different subgroups may change if the sample size was expanded. Second, not all fetal CGIOs were confirmed, and some misdiagnosed cases were included in our study. We should accept that these factors might influence the rates of chromosomal abnormalities to a certain extent. Finally, not all women with CGIO underwent amniocentesis or cordocentesis, which might be relevant from the perspective of selection bias. Some results found in our study also differed from previous reports. The abnormal genetic results found in our study may not represent the majority of CGIO abnormalities but can also expand the genetic scope of CGIO to some extent. In the future, more cases need to be collected, likely in cooperation with multiple centers, to conduct further statistical analysis and to generate a more complete summary of the findings.

## Conclusion

In summary, our study showed that the risk of pathogenic chromosomal abnormalities and CNVs was increased in the complicated CGIO group compared to that in the isolated CGIO group, especially with DO and anorectal malformation. CNC-seq was recommended to detect submicroscopic chromosomal aberrations for DO and anorectal malformation with a normal karyotype, as the information derived can provide additional clinically relevant information. The relationship between genotypes and phenotypes needs to be explored in the future to facilitate prenatal diagnosis of fetal CGIO and provide new clues into their etiologies.

## Data Availability

The datasets generated and/or analyzed during the current study are not publicly available due to the pregnancies who participated in the study did not agree to use their individual data for publicity in the research, but are available from the corresponding author on reasonable request.

## Abbreviations

CGIO: Congenital gastrointestinal obstruction
NGS: Next-generation sequencing
CNV-seq: Copy number variation sequencing
DO: Duodenal obstruction
SD: Standard deviation
BMI: Body mass index
TOP: Termination of pregnancy
pCNV: Pathogenic copy number variants
VOUS: Variant of uncertain significance
OMIM: Online Mendelian Inheritance in Man
CNV: Copy number variant
